# Barriers to Health Care for Chinese in the Netherlands

**DOI:** 10.1155/2011/635853

**Published:** 2011-07-07

**Authors:** Cha-Hsuan Liu, David Ingleby, Ludwien Meeuwesen

**Affiliations:** Faculty of Social and Behavioural Sciences, Utrecht University, 3508 TC Utrecht, The Netherlands

## Abstract

This study examines utilisation of the Dutch health care system by Chinese people in the Netherlands as well as their attitudes to the system, paying special attention to mental health. Information was gathered by semistructured interviews (*n* = 102). The main issues investigated are access, help-seeking behaviour, and quality of care. Results showed that most respondents used Dutch health care as their primary method of managing health problems. Inadequate knowledge about the system and lack of Dutch language proficiency impede access to care, in particular registration with a General Practitioner (GP). Users complained that the care given differed from what they expected. Results also showed that the major problems are to be found in the group coming from the Chinese-speaking region. Western concepts of mental health appear to be widely accepted by Chinese in the Netherlands. However, almost half of our respondents believed that traditional Chinese medicine or other methods can also help with mental health problems. The provision of relevant information in Chinese appears to be important for improving access. Better interpretation and translation services, especially for first-generation migrants from the Chinese-speaking region, are also required.

## 1. Introduction

The Chinese population in the Netherlands, as in many other countries, has increased greatly in the last ten years. This population is currently approaching 100,000 [1, 2], making it the fourth largest ethnic minority in the country as well as one of the longest established. In this paper we include in the category “Chinese” not only persons originating from the Chinese-speaking region (mainland China, Hong Kong, Macau, and Taiwan), where Chinese culture is dominant, but also those coming from overseas Chinese communities in, for example, Indonesia or Surinam. We also include children of migrants who were born in the Netherlands, that is, the second generation.

Traditionally, Chinese have had a reputation for keeping themselves to themselves; they are often assumed to solve their problems within their own community. Language barriers have also hampered contact with Dutch society [1, 3]. For all these reasons, Chinese remain an invisible minority to most Dutch people, and up to now little research has been done on them, in particular regarding their health. This paper reports an investigation into the attitudes of this group towards the Dutch health care system and the factors influencing their willingness to make use of it. 

In the Dutch health care system, the general practitioner (GP) functions as a “gatekeeper” to specialist services [4]. Our main interest in this study was in mental health care, but since this is fully integrated within the general health care system and is only accessible through the GP, we also asked questions about health care in general. 

Mental health services in the Netherlands are financed from the social health insurance system, in which participation is compulsory for all residents. For some treatments, a partial contribution from the patient is required. Outpatient services are provided by a network of community mental health care centres, backed up by inpatient services.

Research in many countries has shown that Chinese people are less likely than other ethnic groups to utilise mainstream health services and has identified some of the barriers to uptake [5–12]. In the Netherlands, however, very little is known about the use of health services in general, and mental health care in particular, by this group. Geense [13] and Liu et al. [1] reported that, while it is unclear whether Chinese use mental health care less than other ethnic groups, there are indications that care delivery for them is far from optimal. To be able to provide more appropriate care for this group it is first necessary to understand the factors which may impede their use of the existing services. 

The aim of this exploratory study was therefore to gain insight into the attitudes of Chinese in the Netherlands to the Dutch health care system, paying particular attention to mental health. What factors influence their willingness to make use of the system? What are their beliefs concerning mental health? Information was gathered by semistructured interviews. Before describing the study we will briefly discuss the main issues it deals with: access, help-seeking behaviour, and quality of care.

### 1.1. Access

Access to health services has two main ingredients: *entitlement* to use the services and the *accessibility *of services in terms of how easily they can be located and how many barriers to their use are experienced. Entitlement to use Dutch health services is restricted to legal residents who have paid the compulsory health insurance contributions. Undocumented migrants, although not allowed to join the insurance system, may receive government-subsidised health care if they are unable to pay costs themselves. However, many appear not to know this.

Accessibility can be broken down into several components. To start with, people must identify themselves as having a problem that can be helped by the available services. Differences in health-seeking behaviour may thus result from divergent beliefs concerning illnesses, their causes, and treatment; Kleinman's concept of “explanatory models” [14] was developed to explore such beliefs. Explanatory models among Chinese may be strongly influenced by traditional Chinese medicine. Secondly, people need knowledge about the health care system and skills for obtaining help from it (health literacy). For example, those who are unfamiliar with the system may have difficulty getting past gatekeeper agencies such as general practitioners, resulting in overutilisation of crisis or emergency services [15, 16]. 

Another important barrier to access is lack of *trust*. If people do not trust the services, they will be inclined to seek help only when absolutely obliged to—for example in an emergency or in advanced stages of illness [17]. They may suppress or hide their problems, resort to traditional healers and self-medication, or return to their home country for treatment [18, 19]. 

Perhaps the most serious barriers to access are formed by communication problems [20]. Unless health services provide effective ways of overcoming such problems they can lead to inaccurate diagnoses, noncompliance with treatment, and inappropriate use of services [1, 16, 21]. It is important that both parties understand not only each other's words but also their perspectives and expectations.

### 1.2. Help-Seeking Behaviour

Help-seeking behaviour will be influenced by the barriers to access which migrants encounter. Chinese in the USA and UK show several different patterns of health-seeking behaviour [22–24]: either self-treatment and home remedies, combinations of Western and traditional health services, or exclusive utilisation of either Western or traditional Chinese treatments. 

Regarding mental health, Fang and Schinke [25] found that a high percentage (84%) of Chinese migrants in the USA attending a community mental health service used complementary therapies. Research on Chinese migrants in British Columbia has reported that demographic characteristics (age, place of origin, educational level, and marital status) influence the utilisation of mental health care [10]. Chen et al. [12] found an association between language proficiency and mental health care utilisation. Chung [26] mentioned shame and stigma as important barriers to help-seeking, while Fung and Wong [9] suggested that explanatory models of mental illness and the perceived availability of appropriate services determined the readiness to use mental health services. 

In the Netherlands, Liu et al. [1] found that language barriers and lack of knowledge about the services available were major factors discouraging Chinese from using mental health services. Other cultural barriers were the pervasive stigma attached to mental health problems, differences in communication style, the tendency to conceal problems, different ideas about appropriate help, and distrust of mental health care professionals. 

Hsiao et al. [23] suggested that Chinese-Americans lacking English proficiency were more likely to use complementary and alternative medicine than Chinese-Americans who were proficient in English. At the same time, Chinese-Americans who immigrated more than 10 years ago were less likely to use complementary medicine alongside Western health care than Chinese-Americans who were born in the USA. Like Ying and Miller [27], these researchers suggested that acculturation was an important predictor of help-seeking behaviour. In the present research, we examined the effect of length of residency in the Netherlands and three other acculturation-related factors: self-labelling of ethnicity, Dutch language proficiency, and social contacts with Dutch people.

### 1.3. Quality of Care

The perceived quality of available health services is another factor influencing the readiness of users to seek help [28]. Research into the quality of health care for migrants and ethnic minorities [16, 29, 30] studies its effectiveness in terms of outcomes, the satisfaction of both users and health care workers, and the extent to which the treatment process was properly carried out, avoiding therapy noncompliance and dropout. All these aspects of good care are undermined by poor communication [31, 32]. Clients with a migrant background are often perceived by professionals as making inappropriate, incoherent, or ill-formulated requests, while from the point of view of these clients the professional listens poorly, lacks insight into the problem, and proposes inappropriate or irrelevant solutions [33]. 

Using the concepts discussed above, the following research questions were formulated: What is the respondents' level of acculturation? How easy is their access to health care? What are their help-seeking tendencies? What are their attitudes to Dutch health care and to issues concerning mental health?

## 2. Methods

### 2.1. Subjects and Procedure

The present study can be characterised as “mixed-methods” research because quantitative data were supplemented by qualitative data from open-ended questions. We examined both the statistical associations of behaviour and attitudes and the reasons or explanations given by respondents. The semistructured questionnaire used in this study was prepared in both Chinese and Dutch versions and contained six sections: demographics, acculturation, access to health care, help-seeking tendencies, opinions about Dutch health care, and mental health issues. Before use, the questionnaire was tested and fine-tuned in a pilot study with 10 Chinese respondents. 

Five interviewers were employed (including the researcher), each of whom was proficient in at least one of the following: Dutch, Mandarin, and Cantonese. In this way it was possible to interview all respondents in their preferred language or dialect. Although the questionnaire was self-administered, the interviewers were available to assist the respondents with difficulties in understanding or answering the questions. 

The sample consisted of Chinese residing in the Netherlands and originating from the Chinese-speaking region (defined here as mainland China, Hong Kong, and Taiwan) or overseas Chinese communities. The latter group are recognised by the Council of the Chinese Minority in the Netherlands (Inspraakorgaan Chinezen) as members of the Chinese minority in the Netherlands [34].

Respondents were recruited in shopping areas of “Chinatowns” or in the vicinity of large Chinese supermarkets in Amsterdam, The Hague, Rotterdam, and Utrecht (the cities in which half Chinese in the Netherlands live [35]). These areas are visited by Chinese people, even those living in other parts of the Netherlands, for shopping and social events. Interviewers approached potential respondents on the street and 53% were willing to cooperate (*n* = 102). To ensure a reasonably representative sample, researchers approached equal numbers of men and women and aimed at a wide age range. Each interview took 10 to 15 minutes and data were collected anonymously. 

### 2.2. Measures

The topics covered in the six sections of the interview are described here in more detail. 

#### 2.2.1. Demographic Information

Background variables included age, gender, civil status, education, region of birth, mother tongue, migration generation, reason for migration, age of migration to the Netherlands, and length of residence.

**Civil Status**

This comprised five categories: married, partnered, single, separated, and widowed. This was recoded as “partnered” (including married or partnered) and “not partnered” (single, separated, or widowed).

**Education**

This was determined by the highest education completed either in the Netherlands or in the region of origin. The answers were grouped into three categories: (1) primary school or lower, (2) secondary or lower vocational education, and (3) higher education.

**Region of Birth**

This included 9 categories (China, Hong Kong, Taiwan, the Netherlands, Indonesia, Suriname, Malaysia, Singapore, and “other regions”). China, Hong Kong, and Taiwan are defined as the Chinese-speaking region, while Indonesia and Suriname are former Dutch colonies. The variable was recoded into four categories: Chinese-speaking region, former Dutch colonies, “other regions,” and the Netherlands.

**Mother Tongue**

This question was open-ended. When the mother tongue of the respondent was Chinese, details of the dialect were asked for.

**Migration Generation**

Two groups were formed: (1) first generation (born outside the Netherlands) and (2) second generation (born in the Netherlands). None of the respondents were from the third or later generations.

**Reason for Migration**

Answers to this open-ended question were grouped into five categories: family reunification or formation, economic migration, study, political factors, and “other reasons.”

**Length of Residence in the Netherlands**

This was measured in years.

#### 2.2.2. Acculturation Factors

Three aspects of acculturation were measured: self-labelling of ethnicity, Dutch language proficiency, and social contacts with Dutch people.

**Self-Labelling of Ethnicity**

Respondents were asked which ethnicity they used to describe or introduce themselves to other people. Answers were coded as Chinese, Dutch, mixed ethnicity, or other ethnicity. Mixed ethnicity could combine Chinese, Dutch, or other ethnicities.

**Dutch Language Proficiency**

Respondents assessed their own proficiency in reading, writing, and speaking Dutch. Answers were coded using a 4-point scale: none (0), poor (1), moderate (2), and good (3). Because of the high degree of intercorrelation between these three variables (*α* = 0.97), a summary variable (Dutch proficiency) was created using the mean of all three.

**Social Contacts**

Two questions were asked: “which ethnic background do most of your friends have?” and “what is the frequency of your contact with native Dutch?” Answers to the first question were coded as Chinese, Dutch, mixed ethnicity, or other ethnicities. The options for the frequency of contact with native Dutch were “seldom,” “sometimes,” and “often,” based on the respondents' self-perception.

A positive correlation was found between the variables “Dutch language proficiency” and “frequency of contact with native Dutch” (*r* = 0.56, *P* < .01). 

#### 2.2.3. Access to Health Care

Questions on this subject related to entitlement, accessibility, and utilisation of Dutch health care.

**Entitlement**

Respondents were asked if they had health insurance. If the answer was “no,” interviewers asked what the reason was.

**Accessibility of Dutch Health Care**

Two items were included: (1) whether respondents had received information about the Dutch health care system and (2) whether they were registered with a general practitioner (GP).

**Utilisation of Dutch Health Care**

Respondents were asked whether they had ever used Dutch health care.

#### 2.2.4. Help-Seeking Tendency

Respondents were asked which form of care they usually used for regaining health. The options were: Dutch health care, traditional Chinese medicine, both of these, or other kinds of care. Respondents who used other kinds of care were asked to give further details. A new variable “tendency to seek help from the Dutch care system” was made, contrasting positive attitudes to seeking help from the Dutch system (whether or not in conjunction with other forms of treatment) with negative ones.

#### 2.2.5. Opinions about Dutch Health Care

Respondents were asked whether or not they had difficulties in using Dutch health care. An open-ended question asked for their opinions about Dutch health care and the ways in which it could be improved for Chinese migrants.

#### 2.2.6. Mental Health Issues

This section comprised three questions: (1) is Dutch (Western) health care helpful for problems related with mental health? (2) Are there other ways of dealing with mental problems? (3) What kind of care would you suggest for family or friends who have mental problems? The response alternatives were “yes,” “no,” and “do not know/not applicable.” Respondents were asked to give further details to clarify their answers.

### 2.3. Analysis

Relationships between the quantitative variables were examined using parametric and nonparametric tests of bivariate association. Because of the moderate sample size, only limited multivariate analyses could be used. In the presentation of results only significant differences will be mentioned.

## 3. Results

### 3.1. Demographics and Migration Background

Table 1 shows the characteristics of the research sample. Four groups are identified: three first-generation groups (born in the Chinese-speaking region, former Dutch colonies, or other regions) and the second-generation group (born in the Netherlands). Eighty-two percent of respondents belonged to the first generation and 18% to the second. The majority (69%) came from the Chinese-speaking region. 

Respondents' ages varied widely, from 17 to 79 (*M* = 39, SD = 16). The mean age of the four groups varied considerably; the average age of migrants from former Dutch colonies was 59, while second-generation Chinese who had grown up in the Netherlands were less than half as old. Women comprised the majority of the latter two groups, while the second generation was better educated than the first. Comparing these data with figures for the Dutch population revealed an increased proportion with the lowest and the highest levels of education, with fewer in between (*X* ^2^ = 28.11, *df* = 3; *P* < .01). 

For most respondents (83%) Chinese was their mother tongue. Five different dialect groups were spoken: Mandarin (official spoken Chinese), Yue (Cantonese), Wu, Hakka, and Min. Other mother tongues were Dutch (11%) and Indonesian (5%). 

About half the respondents (47%) had lived in the Netherlands for more than 20 years. People who had lived in the Netherlands for less than five years comprised 18% of the research group. The main reasons for migration were family reunion or formation (48%) and economic migration (24%). Four percent had migrated because of the political situation in their country of origin. 

Half of those arriving since 2000 came in order to study. None of those who migrated to the Netherlands before that year came for this purpose. The average educational level of those arriving since 2000 was also considerably higher than that of earlier migrants (means: 2.39 versus 1.79, *t*  (79) = 3.85, *P* < .001). These findings reflect a marked change in the character of recent Chinese migration to the Netherlands.

### 3.2. Acculturation Factors

Most respondents born in the Chinese-speaking region described their own ethnicity as Chinese (91%), while most born in the Netherlands or former Dutch colonies described it as mixed (42% and 52%). Whereas 82% of the respondents from the first generation referred to themselves as Chinese, only 20% from the second generation did so. 

Considerable differences in mean Dutch proficiency scores were found between the different regions in which respondents were born, ranging from 1.16 for the Chinese-speaking region to 2.94 for the Netherlands. Respondents from former Dutch colonies scored almost as well (2.62) as those born in the Netherlands. The 5 respondents born in other countries also had fairly high scores (2.27). The scores of respondents born in the Netherlands were significantly higher than those born in the Chinese-speaking region (*t*  (85.9) = 14.8, *P* < .001).  Table 2 shows the intercorrelations between the variables relating to demographics, acculturation factors, and utilisation of health care.

What determines the level of Dutch language proficiency among the group born in China? Stepwise multiple regression analysis showed that gender, educational level, age, and the length of time people had been living in the Netherlands did not significantly affect language proficiency. A higher age on arrival in the Netherlands, as well as coming to the Netherlands for purposes of study, had a negative influence on Dutch language proficiency; the frequency of contact with Dutch had a positive influence. The main determinant was the age at which respondents had migrated to the Netherlands (*β* = −.047, *P* < .001). The second most important factor was whether they had come for study or for other purposes. Students appeared to make little effort to learn Dutch, perhaps because they did not expect to stay in the country (*β* = −.800, *P* < .001). 

Finally, the frequency of contact that respondents had with native Dutch also increased their language proficiency (*β* = .329, *P* = .002), though this influence was probably in both directions. These results should, however, only be regarded as tentative, as the sample on which they are based (*N* = 70) is relatively small.

### 3.3. Access to Health Care

#### 3.3.1. Entitlement

Only seven respondents had no health insurance. All were men who had migrated from the Chinese-speaking region since 1990. Compared to other men in this category, they were less well educated (*t*  (42) = −2.00, *P* = .05). One of them mentioned financial reasons for not taking out insurance, while another considered insurance unnecessary because he seldom used Dutch health care. The remaining five were undocumented and not allowed to take out insurance.

#### 3.3.2. Accessibility of Dutch Health Care

Knowledge of the Dutch health care system: less than half of the respondents (46%) had received information about how to use the Dutch health care system. Sources of information included health insurance companies, health care providers (including municipal health centres), official brochures, school, work, or the media.

 Female respondents were significantly more likely than males to report having received information (56% versus 33%; *X* ^2^ = 5.27, *df* = 1, *P* < .05). Respondents who grew up in the Netherlands or a former Dutch colony were also more likely to have received information than those coming from the Chinese-speaking region or other countries (62% versus 40%; *X* ^2^ = 4.17, *df* = 1, *P* < .05). Interestingly, there were no associations with any acculturation variables.

Registration with a GP: sixteen percent of respondents reported that they had not registered with a GP. Reasons given included lack of insurance, lack of information about how to find a GP and get registered, and no idea about the function of the GP (using emergency care instead). In some cases the workplace or school had organised a clinic centre for primary care.

Those who registered with a GP were slightly more likely to have received information about the use of Dutch health care than those who did not (51% versus 19%; *X* ^2^ = 5.70, *df* = 1, *P* < .05). The Dutch proficiency of those who did not register was very low (0.48 versus 1.88; *t* = −5.33, *P* < .01).

#### 3.3.3. Utilisation of Dutch Health Care

Eighteen percent of respondents (*N* = 18) had never used the Dutch care system. There was a high degree of overlap with the group who had not registered with a GP; however, 21% of those who had registered with a GP had never used health care.

Using the system was more common among elderly people  (*r* = 0.27, *P* < .01), among women rather than men (90% versus 73%; *X* ^2^ = 4.51, *df* = 1, *P* < .05) and people with less education (*r* = −0.22, *P* < .05). People who had been in the Netherlands longer (*r* = 0.42,  *P* < .01), had better Dutch proficiency (*r* = 0.25, *P* < .05), and had received information about the system (82% versus 55%; *X* ^2^ = 7.61, *df* = 1, *P* < .01) were more likely to use Dutch health care as well.

### 3.4. Help-Seeking Tendency

Most respondents (73%) sought help only from the Dutch health care system, 4% used only traditional Chinese medicine, and 13% used both. The other respondents (11%) said they preferred to help themselves, for example, by buying medicines over the counter. There were no significant associations with demographic or acculturation variables. 

A new variable was made contrasting those with positive attitudes to seeking help from the Dutch system (perhaps in conjunction with other forms of treatment) with those who had negative attitudes. Only 15% of the sample had negative attitudes. Their Dutch proficiency was extremely low in comparison with those who had positive attitudes (0.56 versus 1.85, *t*  (25.5) = 6.06, *P* > .001). Moreover, a lower percentage had health insurance (60% versus 99%, *P* < .001 by Fisher's exact test) and was registered with a GP (40% versus 92%, *P* < .001 by Fisher's exact test).

### 3.5. Opinions about Dutch Health Care

A substantial proportion of the respondents (40%) said they had difficulties in using the Dutch care system. They named problems such as language barriers, long waiting times and procedures, diverging health concepts, and discrimination. A few people reported that, due to their lack of Dutch proficiency, GPs did not want to take the time to explain the diagnosis or treatments to them. All respondents who mentioned language barriers had labelled themselves as Chinese, and most of them (71%) originated from the Chinese-speaking region. Respondents from the second generation (*X* ^2^ = 6.31, *df* = 1, *P* < .05) and those with better Dutch proficiency (*r* = −.35, *P* < .01) were less likely to report difficulties.

Seventy-five percent of those who gave yes/no answers believed there was room for improvement in Dutch health care for Chinese. Half of them mentioned the provision of interpretation or translation services. Other suggestions included reducing waiting lists, offering walk-in services, increasing the cultural sensitivity of health workers, and providing information for Chinese people about Dutch (Western) medical concepts. 

Some of those who did not think there is room for improvement said they thought it unlikely that the system would be adapted just for the benefit of a small group of users. A female respondent of Indonesian origin suggested that Chinese health care users should try to improve their Dutch proficiency instead of asking for additional language facilities.

### 3.6. Mental Health Issues

When asked if they had confidence that Dutch (Western) mental health care could help people with mental illness, 20% of the respondents said that they did not know or that the question was not applicable. Of those who did give a definite answer, 79% said “yes.” Second-generation Chinese were more likely to say “yes” (90%) than first-generation ones (76%). 

Sixty-two percent of those giving yes/no answers thought that there are alternative methods of helping with mental problems besides Dutch mental health care. These methods included both traditional Chinese remedies and general ones such as social support. 

Regarding the willingness to recommend seeking help for mental problems (not necessarily from Dutch mental health care), 86% of the 89% who gave a definite answer said they would suggest their relatives or friends seek help if they thought it was needed. Those answering “yes” to this question had a higher level of education than those answering “no” (*t*  (87) = 2.19, *P* < .04).

Fifty-seven percent of respondents had relatives or friends with mental health problems or had themselves experienced issues related to mental health problems in the Netherlands.

## 4. Discussion and Conclusions

This study is set out to examine the utilisation of health care services by the Chinese minority in the Netherlands and this group's attitudes concerning health, paying particular attention to mental health.

Table 1 shows that there are three groups of first-generation migrants, originating from the Chinese-speaking region, former Dutch colonies, and other countries. The latter group was too small for statistical analyses, but there was a clear difference between the first two in terms of age and acculturation variables. Migrants from former Dutch colonies were older and had better Dutch language proficiency than those from the Chinese-speaking region. They were also more likely than the latter group to identify themselves as being of mixed ethnicity. Many of them would have made acquaintance with Dutch language and culture before migrating. 

A fresh wave of young migrants from the Chinese-speaking region, with a higher average level of education, arrived from 2000 onwards. Half of them came for purposes of study. The second generation, born in the Netherlands, had the highest level of education and were mostly very well acculturated. These findings reflect the immigration patterns described by Cheung and Lam [36]. 

### 4.1. Access to Health Care

Data on health care utilisation and attitudes showed that the major problems are to be found in the group coming from the Chinese-speaking region. This group contains all of those with no health insurance, as well as most of those who had received no information about Dutch health care, were not registered with a GP, and did not use the Dutch health system. All these characteristics were associated with low levels of Dutch language proficiency (cf. Liu et al. [1]). This proficiency, in turn, was associated with the age at which migrants had arrived in the Netherlands, their frequency of contact with native Dutch, and whether or not they had come to study.

Lack of information about the Dutch health care system was also a barrier to utilisation. Particularly for newcomers, better provision of information about health and health care in Chinese would appear to be important for improving access. Vogels et al. [3] emphasise that learning Dutch is crucial for the integration of Chinese immigrants.

Despite these problems of entitlement and health literacy, most respondents stated a preference for Dutch health care as their main way of managing health problems. There was no evidence of differences in health-seeking tendencies as a function of age, sex, education level, or length of residence in the Netherlands. 

Nevertheless, 39% of respondents reported difficulties in using the system. These were mainly associated with lack of Dutch proficiency. Language barriers need to be addressed energetically [16, 37]. Chen et al. [12] suggested that language is functioning as an indicator of cultural differences and go on to discuss possible cultural barriers to service uptake. However, the findings we report suggest that the main barrier to access in their study may simply have been lack of language proficiency. 

Many of those affected are relatively old and not well equipped to improve their language skills. Better interpretation and translation services are clearly required; the employment of more Chinese health workers would go some way to reducing both linguistic and cultural barriers. Respondents also complained about long waiting times and discrimination. Waiting lists are a problem that affects everybody in the Netherlands.

### 4.2. Attitudes towards Mental Health Care

It is certainly not the case that Chinese do not recognise the existence of mental illness. Nevertheless, it is known [38] that mental illness is associated with stigma for Chinese people, and this may present a major obstacle to receiving help. In the present study, however, we did not get the impression that mental health problems were heavily stigmatised by our respondents. Most of them seemed to feel comfortable talking with us about mental health and said they were willing to talk about it with relatives and friends. 

Western methods of treating mental illness appear to be widely accepted by Chinese in the Netherlands, as indeed they are in the Chinese-speaking region itself. However, 62% of the respondents who answered the question believed that there are also other ways of dealing with mental health problems. This “health pluralism” is a common phenomenon in developing countries, but it is also found in Western societies, where “alternative therapies” and self-help account for a large proportion of all health expenditure [39]. 

This study suggests that Chinese with a higher level of acculturation—in particular, better Dutch language proficiency—have better access to Dutch health care and make more use of it; however, this does not necessarily mean that they abandon a belief in traditional Chinese medicine or other forms of help. This is in line with the US study of Hsiao et al. [23] and the British study of Ma [22], which showed that acculturated Chinese mostly drew upon two medical systems, conventional medicine and traditional Chinese medicine.

### 4.3. Limitations of This Study

In this study it was not possible to compare Chinese with any other ethnic groups. Nor was any information collected on the nature or prevalence of health problems (mental or otherwise). 

The recruitment of respondents on the streets of Chinatowns frequently visited by Chinese for daily shopping and social events may have deprived us of the opportunity to gather ideas from people working during the daytime, especially those working in the restaurant business. In addition, it will have led to under representation of those who do not visit Chinatowns, who may be more acculturated than those who do. 

Finally, although the sample size was large enough to reveal many significant effects, a larger sample would make it possible to use more advanced multivariate analyses (e.g., path analysis) in order to disentangle the relationships among variables. In-depth qualitative studies of how Chinese deal with their mental health problems are also required in order to shed more light on the question of how to provide more accessible and appropriate services for this group.

### 4.4. Conclusion

Despite its limitations, the present study shows that access to health care for Chinese in the Netherlands is closely linked to their proficiency in Dutch. The “Chinese community” comprises several different populations with different demographic and cultural characteristics. The group with the greatest problems of access to health care are those who have migrated from the Chinese-speaking region during the last two decades. 

Cultural differences in relation to health certainly exist, but a belief in Chinese traditional remedies does not necessarily form a barrier to using Dutch care. A lack of cultural competence among health care workers, on the other hand, does. Barriers were not confined to mental health care services but concerned access to health care in general. 

For migrants with a low level of Dutch proficiency, better interpretation and translation services are urgently required; the employment of more Chinese health workers would help to improve both access and the quality of care. Our results suggest that special measures to overcome language barriers need to be taken with migrants from the Chinese-speaking region who arrive later in life, those who seldom have contact with native Dutch, and students not intending to stay permanently. Finally, to overcome the lack of knowledge about health care, activities to improve health literacy are clearly needed, carefully targeted, and adapted so as to have maximum impact on the groups who need them the most [40].

## Figures and Tables

**Table 1:** Characteristics of different subgroups.

		First generation			Second generation	
		Chinese-speaking region* (*N* = 70)	Former Dutch colonies (*N* = 9)	Other (*N* = 5)	The Netherland (*N* = 18)	Whole sample (*N* = 102)
Demography	Mean age	40	59	44	28	39
	% female	50	78	60	67	56
	% partnered	39	56	40	89	49
	Education (1–3)	2.0	2.1	2.2	2.5	2.1
	% mother tongue: Chinese	100	11	60	61	83

Acculturation factors	Dutch proficiency (0–3)	1.2	2.6	2.3	2.9	1.7
	Contact with Dutch (1–3)	2.2	3.0	2.6	2.9	2.4

Access	% with health insurance	90	100	100	100	93
	% received information about health care	40	56	40	67	46
	% registered with GP	79	89	100	100	84

Utilisation	% used Dutch health care	77	89	100	94	82
	% seek help Dutch care	80	100	100	94	85

Opinions on health care	% difficulty using care	49	11	40	17	40
	% thinks room for improvement**	82	60	100	50	75

Attitudes towards mental health	% confidence Mental Health care**	77	75	80	89	79
	% belief in alternatives**	60	50	60	75	62
	% recommend Mental Health care**	84	100	80	88	86

*Includes China, Hong Kong, and Taiwan, **Percentage of saying “yes” among the respondents who gave a “yes/no” answer.

**Table 2:** Correlations (Pearson) between variables concerning demographics, acculturation, and utilisation of health care (*N* = 102).

Variables	Age	Gender	Partnered	Education	Migration generation	Age on arrival in NL	Dutch proficiency	Contact with Dutch	Information received about Dutch care	Family doctor	Use of Dutch care	Difficulty in using Dutch care
Age	1.00											
Gender (1 = M, 2 = F)	.13	1.00										
Partnered (0 = no, 1 = yes)	.44**	−.08	1.00									
Education	−.48**	.04	−.30**	1.00								
Migration generation	−.34**	.09	−.36**	.27**	1.00							
Age on arrival in NL	.58**	.08	.36**	−.20	.32**	1.00						
Dutch proficiency	−.20*	.23*	−.31**	.23*	.59**	−.44**	1.00					
Contact with Dutch	−.40**	.10	−.32**	.30**	.33**	−.34**	.56**	1.00				
Information received about Dutch health care	.07	.23*	.00	.04	.19	.15	.21*	.08	1.00			
Registration with a family doctor (GP)	.17	.21*	.01	−.12	.21*	−.09	.47**	.16	.24*	1.00		
Use of Dutch care	.27**	.21*	.11	−.22*	.10	.02	.25*	.02	.27**	.51**	1.00	
Difficulty in using Dutch health care	.30**	.02	.28**	−.08	−.25*	.34**	−.35**	−.32**	.10	−.12	.04	1.00

**P < .01 (2-tailed).*P < .05 (2-tailed).
